# Adrenal Gland Metastasis Is an Unusual Manifestation of Endometrial Cancer

**DOI:** 10.1155/2013/428456

**Published:** 2013-08-06

**Authors:** Syeda Sadia Zaidi, Vipul T. Lakhani, Oluwole Fadare, Dineo Khabele

**Affiliations:** ^1^Department of Endocrinology, Diabetes and Metabolism, Vanderbilt University Medical Center, Nashville, TN 37232, USA; ^2^Department of Pathology, Microbiology, and Immunology, Vanderbilt University Medical Center, Nashville, TN 37232, USA; ^3^Department of Obstetrics and Gynecology, Division of Gynecologic Oncology, Vanderbilt University Medical Center, B1100 Medical Center North, Nashville, TN 37232, USA

## Abstract

This case report describes a woman treated for stage 1 B grade 3 endometrial adenocarcinoma with surgery and adjuvant radiation therapy who presented 6 months later with pain and symptoms of adrenal insufficiency. A large right adrenal mass revealed adenocarcinoma consistent with the endometrial primary.

## 1. Introduction 

Metastatic spread of endometrial cancer to the adrenal gland is rare. We found only four previously reported cases of endometrial cancer with metastases to the adrenal glands. Three of the 4 patients had other sites of metastatic disease at the time of initial presentation. Here, we present a case of stage IB grade 3 endometrial adenocarcinoma diagnosed with an atypical recurrence in the adrenal glands.

## 2. Case Presentation

A 75-year-old woman presented with weight loss, decreased appetite, lower abdominal pain, and postmenopausal bleeding. Her pulse was 76 beats per minute (bpm), and her blood pressure was mildly elevated to 148/88. She weighed 62 kg, and physical examination was unremarkable. Complete blood count (CBC), chemistries, and renal and liver function tests were normal, and a CA125 tumor marker was normal at 5.1 U/mL (normal < 35). Endometrial sampling demonstrated endometrioid adenocarcinoma, grade 3. Chest X-ray was normal. A computed tomography (CT) of the abdomen and pelvis showed hepatic hemangiomas, bilateral adrenal thickening, and a mass in the body of the uterus. The patient underwent surgical staging by total laparoscopic hysterectomy, bilateral salpingo-oophorectomy, and pelvic and paraaortic lymph node dissection for International Federation of Gynecology and Obstetrics (FIGO) stage IB, grade 3, endometrioid adenocarcinoma. A pretreatment CT scan revealed bilateral pulmonary emboli but no clear evidence of metastatic disease. The patient was started on anticoagulation and treated with whole pelvic radiation therapy. 

**Figure 1:**
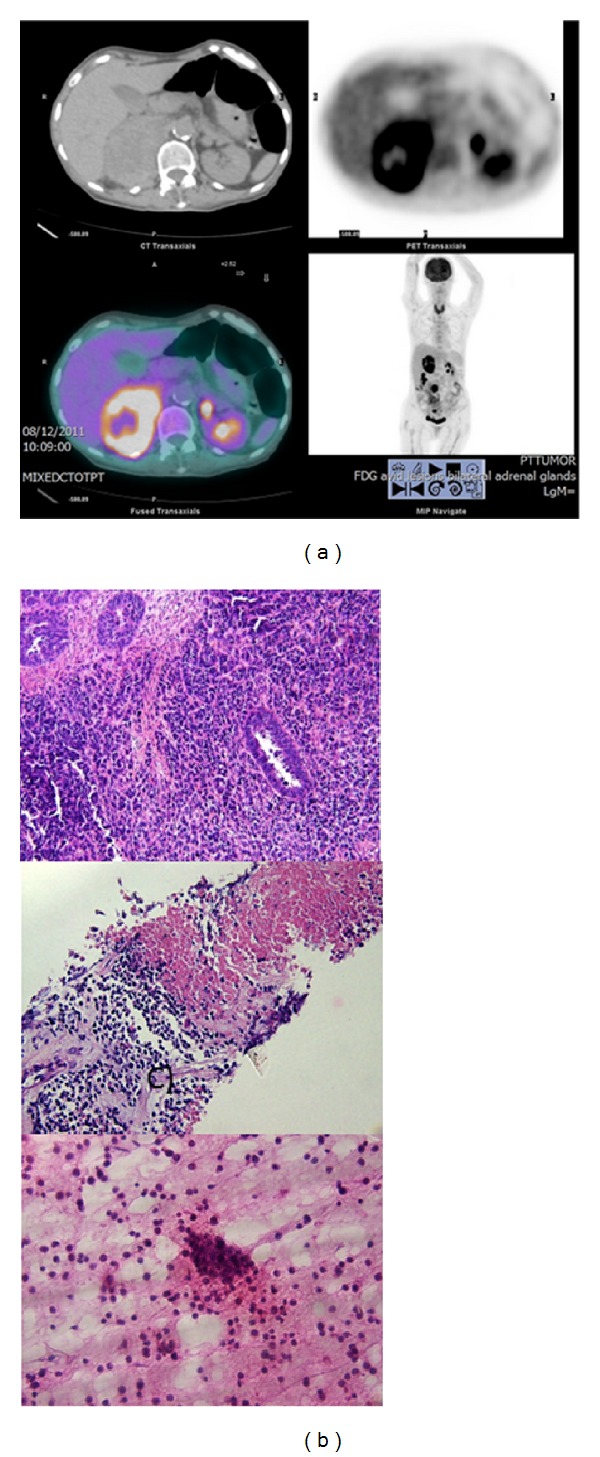
Bilateral adrenal gland metastases from an endometrial cancer primary. (a) Imaging of the adrenal metastases. FDG-PET CT scan images revealed enlarged adrenal glands with increased FDG uptake in both adrenal glands. The upper panels show transaxial CT (left) and FDG-PET (right) images of the adrenals. The lower panel on the left shows a fused CT/ FDG-PET transaxial image, and the lower panel on the right shows a coronal image of the FDG-PET scan. (b) Histological confirmation of the adrenal metastases. The top panel shows a hematoxylin and eosin (H&E) stained image of the primary endometrial tumor (original magnification ×200), with predominantly solid proliferation and scattered endometrial glands. The middle panel is an H&E stain of a core biopsy from the right adrenal gland mass (original magnification ×100) showing viable discohesive tumor cells (lower field) and a large area of confluent tumor necrosis (upper field). The lower panel is an H&E touch cytologic preparation of the right adrenal biopsy (original magnification ×400), showing a cluster of round tumor cells.

Six months after completing treatment, the patient reported right upper quadrant abdominal pain, unexplained weight loss, and nausea. Her pulse was 81 bpm, her blood pressure was 141/93, and her weight had decreased to 57 kg. She had mild right upper quadrant abdominal tenderness on examination. CT imaging showed a new 6.5 × 4.5 cm right adrenal mass, and the left adrenal gland was also mildly prominent. The initial concern was for intra-adrenal hemorrhage, and anticoagulation was discontinued. Laboratory results including CBC, dehydroepiandrosterone sulfate (DHEAS), testosterone, adrenocorticotropic hormone (ACTH) stimulation test, aldosterone, plasma renin activity, and 24-hour urine metanephrine levels were normal. An ACTH level was elevated at 129 pg/mL (range 7–51). A 1 mg dexamethasone suppression test failed to suppress the cortisol levels, and a 24-hour urine cortisol was inconclusive. The decision was made to follow with repeat imaging and blood tests. Fluorodeoxyglucose positron emission tomography (FDG-PET) one month later revealed bilateral adrenal gland masses with intense FDG uptake (Figure 1(a)). The right adrenal mass measured 7.9 × 5.7 cm, and a new left adrenal mass was 1.4 × 1.2 cm. Biopsy of the right side revealed a predominantly necrotic carcinoma consistent with the patient's primary endometrial tumor (Figure 1(b)). A repeat ACTH stimulation test revealed a cortisol level of 21 mcg/dL (range 10.4–26.4), and steroid therapy was initiated with some symptomatic relief. Her CA125 level remained in the normal range at 6.3 U/mL. The patient's rapidly declining physical condition precluded palliative chemotherapy. She was provided supportive care and died 3 months after the diagnosis of the adrenal metastases. 

## 3. Comment

Here, we highlight an unusual case of adrenal metastases from endometrial carcinoma. Despite a relatively normal hormonal evaluation and overlapping symptoms with cancer cachexia and progressive disease, we believe this patient had some evidence of adrenal insufficiency. The prospect that occult adrenal metastases were present at the time of initial diagnosis of endometrial cancer is also a possibility. 

The most common sites of endometrial cancer metastases are the vagina, pelvis, abdomen, and lungs [1]. Metastases to the adrenal glands are rare, with only 4 previously reported cases [2–4]. Three of the 4 reported cases had other sites of metastatic disease. Interestingly, two of the 4 achieved long-term survival after laparoscopic adrenalectomy for isolated recurrences. None of the previously published studies commented on hormonal evaluation. 

A complete hormonal evaluation is critical to exclude pheochromocytoma, primary hyperaldosteronism, and Cushing's syndrome. A typical workup includes resting plasma free metanephrines, plasma renin activity, aldosterone, DHEA-S, and a 1 mg dexamethasone suppression test. Adrenocortical function testing can be performed with a standard ACTH stimulation test, and patients with adrenal metastases should have follow-up testing even if baseline values are normal.

Most adrenal lesions on CT imaging are “incidentalomas” and benign, nonfunctional adenomas. In contrast, adrenal lesions greater than 5 cm with evidence of direct invasion into adjacent tissue and increased size on follow-up imaging are features of malignancy. Pathologic confirmation of malignancy is of prime importance for subsequent management. Collaboration between endocrinology, gynecology, oncology, and endocrine surgery teams should take place to determine the role of biopsy and surgical resection. The diagnosis of pheochromocytoma must be excluded to avoid adrenal and hypertensive crises. Treatment for metastatic tumors to the adrenal glands is based on sites of spread. Adrenalectomy is recommended only if the adrenal gland is the sole site [5]. Laparoscopic adrenalectomy is feasible for small, isolated adrenal metastases and may offer long-term survival in selected patients [3, 4]. 

## 4. Condensation

Metastatic disease to the adrenal glands is an uncommon manifestation of endometrial cancer.

## Abbreviations

ACTH: Adrenocorticotropic hormone
bpm: Beats per minute
CBC: Complete blood count
CT: Domputed tomography
dL: Deciliter
DHEA-S: Dehydroepiandrosterone sulfate
FDG-PET: Fluorodeoxyglucose positron emission tomography
FIGO: International Federation of Gynecology and Obstetrics
H&E: Hematoxylin and eosin
mcg: Micrograms
mL: Milliliter
pg: Pictograms.
